# Diagnosis and Management of Hidden Caries in a Primary Molar Tooth

**DOI:** 10.5005/jp-journals-10005-1415

**Published:** 2017-02-27

**Authors:** Arwa Gera, Uri Zilberman

**Affiliations:** 1 Resident, Department of Pediatric Dentistry, Barzilai University Medical Center, Ashkelon, Israel; 2 Head, Department of Pediatric Dentistry, Barzilai University Medical Center, Ashkelon, Israel

**Keywords:** Computed tomography scan, Deciduous molar, Hidden caries, Preeruptive intracoronal lesion.

## Abstract

Hidden caries is a dentinal lesion beneath the dentinoenamel junction, visible on radiographs. A single report described this lesion in primary dentition. This case report describes a case of hidden caries in a mandibular second primary molar, misdiagnosed as malignant swelling. A 3-year-old white girl was referred to the Department of Pediatric Dentistry with a chief complaint of pain and extraoral swelling on the right side of the mandible for the last 3 months. She was earlier referred to the surgical department for biopsy of the lesion. Radiographic and computed tomography scan examination showed a periapical lesion with buccal plate resorption and radiolucency beneath the enamel on the mesial part of tooth 85. The tooth was extracted, and follow-up of 2 years showed normal development of tooth 45. The main problem is early detection and treatment, since the outer surface of enamel may appear intact on tactile examination.

**How to cite this article:**

Gera A, Zilberman U. Diagnosis and Management of Hidden Caries in a Primary Molar Tooth. Int J Clin Pediatr Dent 2017;10(1):99-102.

## INTRODUCTION

Hidden caries is a dentinal lesion beneath the dentinoenamel junction, visible on radiographs. It is also known as preeruptive intracoronal resorption (PICR), preerup-tive intracoronal radiolucent lesion (PICRL) or preerup-tive caries. The prevalence of PICR in permanent dentition is 2 to 6%, depending on the tooth and radiographic technique. When bite-wings were used, the prevalence is 4% for the mandibular first molar, 2% for the mandibular first premolar, and 1% for the maxillary first molar, maxillary first premolar, and mandibular second molar.^1^ When panoramic radiographs were used, the prevalence is 4% for maxillary first molars, and 3% for mandibular first molars. Usually, a single tooth is affected. However, cases of multiple PICRLs in individual subjects have also been reported.^2^ A single report of PICRLs in primary teeth has been published.^3^ Nearly half of the lesions extend to more than two-third of the dentin.^4^ No association was found between PICR and gender, race, medical conditions, systemic factors, or fluoride supplementation.^1,4,5^

The etiology of PICRL remains a controversy. Suggested causes include apical inflammation of primary molars (relevant only to PICRL in premolars) and dental caries. The most likely hypothesis is that the defects are acquired as a result of coronal resorption. According to Seow et al,^5^ local factors play an important role in the etiology. There is a significantly high association between ectopically positioned teeth and PICRL, which suggests that an ectopic position is a trigger factor. Pressure resulting from an abnormal position may induce sufficient local damage to the tooth’s protective covering causing resorp-tive cells to enter through the enamel. Loss of the integrity of the reduced enamel epithelium may allow osteoclasts, multinucleated giant cells, and chronic inflammatory cells to enter the tooth and initiate resorption of dentin.^5^

This report describes a case of PICRL in a mandibular second primary molar and the subsequent treatment.

## CASE REPORT

A 3-year-old white girl was referred to the Department of Pediatric Dentistry at Barzilai University Medical Center, Ashkelon, Israel, with a chief complaint of pain and extra-oral swelling on the right side of the mandible for the last 3 months. Her medical history was not remarkable. She attended several pediatric dentists for diagnosis, who prescribed antibiotic therapy. The swelling regressed and reappeared. Later, she was referred for a panoramic X-ray, misdiagnosed as malignant swelling, and scheduled for biopsy at oral surgery.

**Fig. 1:**
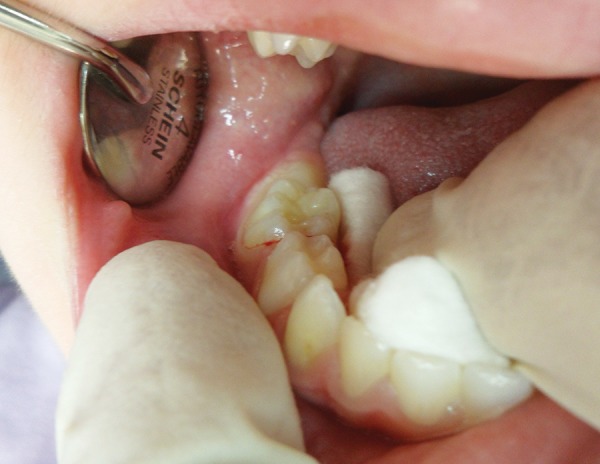
Intraoral examination at the first visit. Note: Intact primary dentition with buccal cortical plate expansion on the right side of the mandible

Extraoral examination revealed swelling on the right side of the mandible, which was diffuse, tender, and warm to palpation. The overlying skin appeared flushed. Intraoral examination revealed intact primary dentition with buccal cortical plate expansion on the right side of the mandible (Fig. 1).

**Fig. 2:**
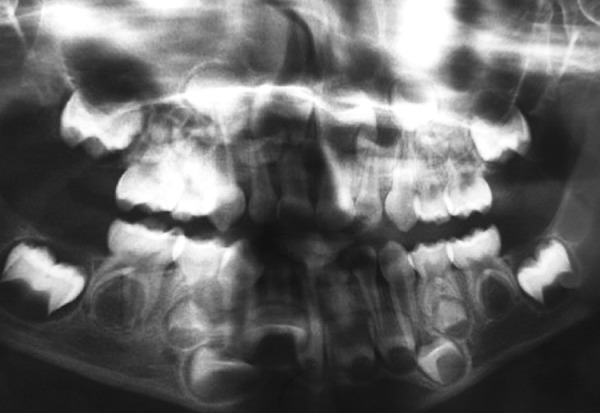
Panoramic radiograph. Note: Periapical radiolucency at tooth 85

**Fig. 3:**
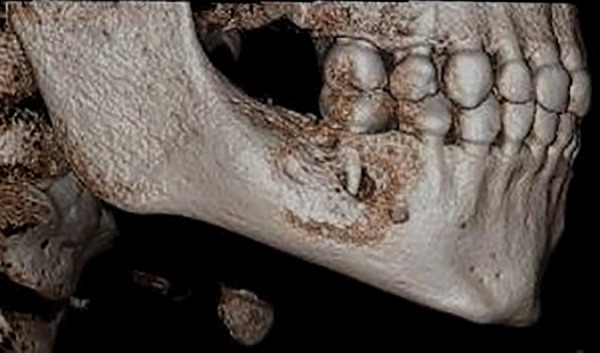
Computed tomography scan of right lower area. Note: Extensive bone resorption of the buccal cortical plate of tooth 85 with the MB root being in contact with the oral soft tissues

**Fig. 4:**
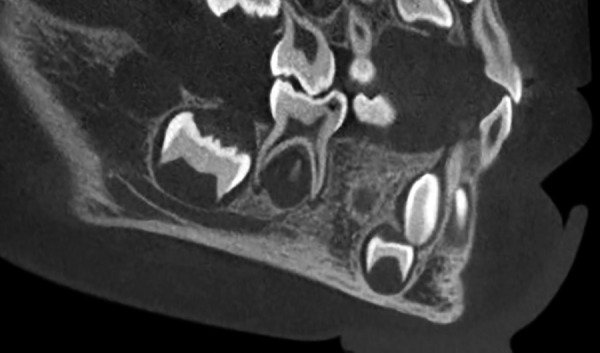
Computed tomography scan. Note: A radiolucent area in the crown extending from the occlusal surface into the dentin under the MB cusp

On panoramic radiograph, tooth 85 had a periapical radiolucency (Fig. 2). The computed tomography scan revealed bone resorption of the buccal cortical plate of tooth 85 with the mesiobuccal (MB) root being in contact with the oral soft tissues and a radiolucent area in the crown extending from the occlusal surface into the dentin under the MB cusp (Figs 3 and 4). Preeruptive intracoro-nal resorption was diagnosed, and the decision was made to extract the tooth.

**Fig. 5:**
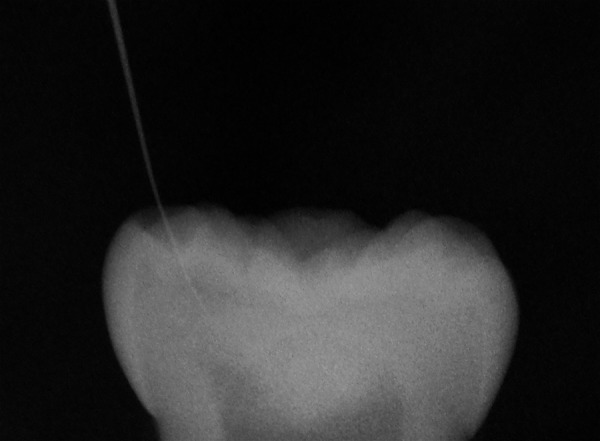
X-ray of tooth 85 after extraction. Note: The exterior opening of the hidden caries was located using a #8 file that entered from the mesial central fissure reaching the pulp horn

Local anesthesia was applied and tooth 85 was extracted. The exterior opening of the hidden caries was located using a #8 file that entered from the mesial central fissure and reached the pulp horn (Fig. 5).

**Fig. 6:**
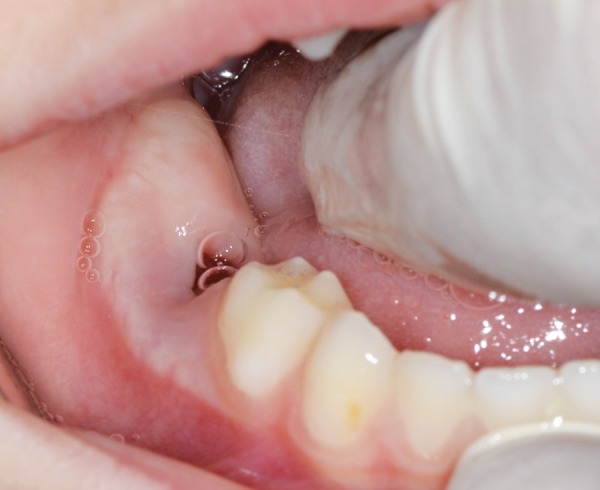
Follow-up examination after 1 week. Note: Proper soft tissue healing

**Fig. 7:**
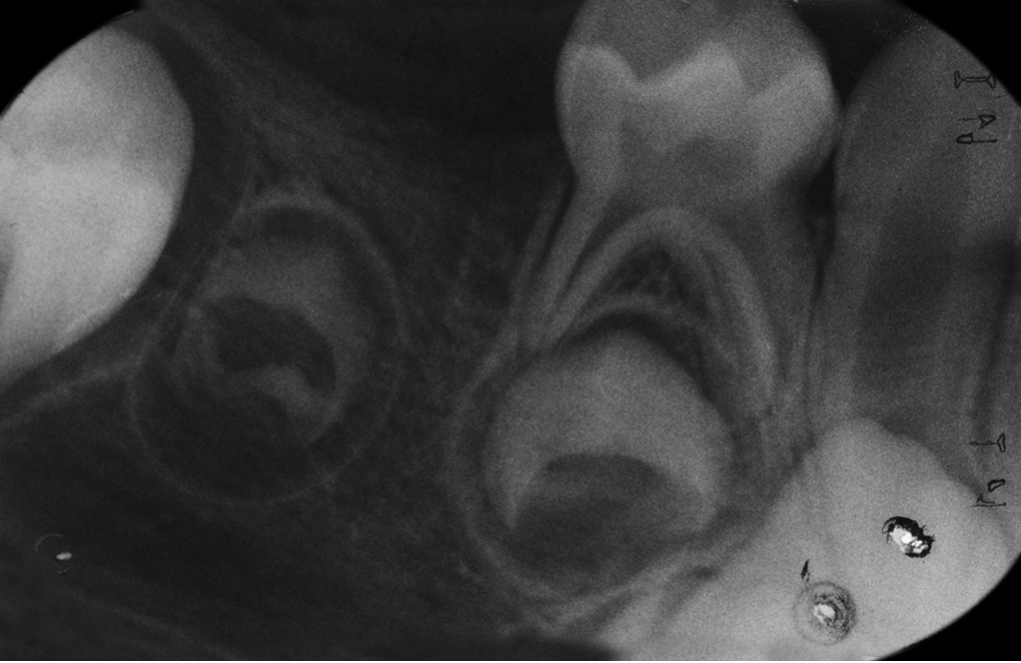
Periapical radiograph, 6 months after the extraction

**Fig. 8:**
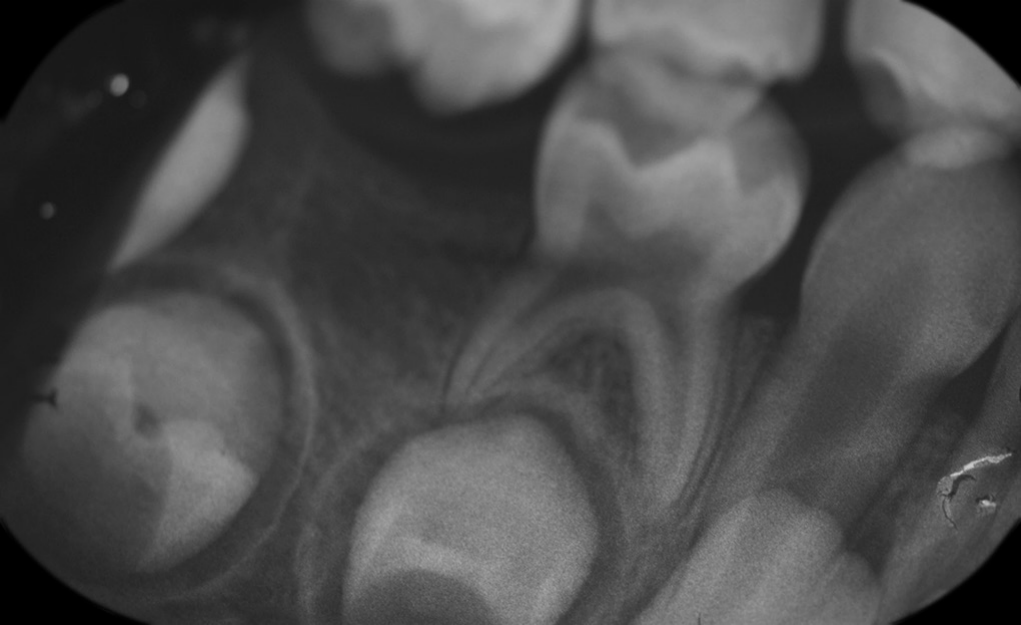
Periapical radiograph, 2 years later. Note: Normal development of the succedaneous tooth

Follow-up examination 1 week later showed soft tissue healing. 6 months later, no clinical symptoms or swelling were observed (Figs 6 and 7). Two years later, radiographic findings showed normal development of the succedaneous tooth (Fig. 8).

## DISCUSSION

This case demonstrated a misdiagnosed PICR of a man-dibular second primary molar, as malignant swelling.

The purpose of this report is to increase the clinician’s awareness and clinical surveillance regarding diagnosis of PICRL, and to avoid further misdiagnosis (artifacts/malignancy). The management of PICRL in permanent dentition varies and may consist of immediate surgical exposure^5^ with curettage of the defect or to wait for tooth eruption to achieve occlusal access for restoration of the defect.^6^ In this case of a primary molar, extraction was the treatment of choice due to large periapical lesion reaching the underlying permanent tooth follicle and very young age.

Clinical and histological evidence from several case reports has suggested that these lesions are likely to be resorptive in nature.^2-4,7,8^ Although the etiology and factors associated with the initiation of resorption remain unknown, resorptive cells originating from the surrounding bone are thought to enter the dentin through a break in the dental follicle and enamel or cementum.^3,9^

According to Kraus and Jordan,^10^ calcification may begin as early as 18 weeks on the tip of the MB cusp, while the other cusps calcify later (mesiolingual—23 weeks, distobuccal—26 weeks, distal or distolingual—28 weeks). At 28 weeks, all five centers of calcification are present, and only then the intercuspal coalescence occurs, and varies with respect to location. Union between the MB and distobuccal cusps may occur via their distal and mesial cusp ridges respectively (at 30 weeks), or the mesial marginal ridge may be the first to calcify (at 30 weeks), thereby connecting indirectly, or in a roundabout way the two mesial cusps. Whichever union occurs first, it is followed by the alternate coalescence to form a calcified ring uniting distobuccal, MB, and mesiolingual cusps (at 32 weeks). Coalescence is complete by 36 weeks, and a small area of uncalcified tissue remains in the middle of the occlusal surface on the distal half of the crown. The 36-week mandibular second molar crown features sharp conical cusps, angular ridges, and a relatively smooth occlusal surface, indicating that considerable deposition of enamel is yet to occur before the completion of calcification.

Based on this evidence, hidden caries may be due to a faulty union or imperfection at the time of coalescence of the centers of calcification. This suggests that occlusal surface defects on molar crowns are intimately associated with the early calcification pattern peculiar to the individual teeth.

## CONCLUSION

Increased awareness of this entity may improve diagnosis, allow early treatment, and avoid misdiagnosis.
